# Phytochemical screening and antidiabetic effects of fruit rind of *Momordica dioica* roxb. on streptozocin induced type 2 diabetic rats

**DOI:** 10.1016/j.heliyon.2022.e08771

**Published:** 2022-01-13

**Authors:** Md. Mynul Hassan, Shihab Uddin, Amrita Bhowmik, Ayesha Ashraf, Md. Mahmodul Islam, Begum Rokeya

**Affiliations:** a Department of Biotechnology and Genetic Engineering, Khulna University, Khulna, Bangladesh; b Department of Biochemistry and Molecular Biology, Jahangirnagar University, Savar, Bangladesh; c Department of Applied Laboratory Sciences, Bangladesh University of Health Sciences, Dhaka, Bangladesh; d Biotechnology and Genetic Engineering Discipline, Khulna University, Khulna, Bangladesh; e Department of Pharmacy, Daffodil International University, Dhanmondi, Dhaka, Bangladesh; f Department of Pharmacology, Bangladesh University of Health Sciences, Dhaka, Bangladesh

**Keywords:** *Momordica dioica*, Diabetes, Streptozocin, Serum glucose, Cholesterol

## Abstract

*Momordica dioica (M. dioica)* is a gourd like blooming plant that is readily available in Bangladesh, requiring biological research to discover its therapeutic values. The goal of our research was to see if the ethanolic extract of this plant had any anti-hyperglycemic properties. Water, glibenclamide and *M. dioica* extracts were fed to Streptozocin induced type-2 diabetic rat models at a dose of 1.25 g/kg body weight (bw) for 28 days to see what kind of effects they had on serum glucose, insulin, liver glycogen and lipid contents. Except for the control group, all the groups followed a pattern of maintaining the body weight. The oral glucose tolerance test was observed to be improved in extract after 14 days of the experiment. When assessed with the control group, the *M. dioica* extract showed a significant (*p* = 0.0015) decrease in postprandial serum glucose level (M±SD, mmol/l, 13.23 ± 1.03 control Vs 11.47 ± 2.21 extract) at 120 min. The treatment of diabetic model rats with extract resulted in a 7% (p < 0.0001) reduction in serum cholesterol levels. While subsequent 28 days of treatment, insulin levels were found to be lowered in all groups (from 246.76 to 200.44 pg/dL; p < 0.0001 for standard and from 309.01 to 204.61 pg/dL; p < 0.0001 for sample). The results revealed that prolonged administration of *M. dioica* improved the glycemic and lipidemic state of type-2 diabetic rats, implying that more research is needed to identify the active ingredient (s).

## 1 Introduction

Diabetics are a category of metabolic disorders in which the body no longer generates insulin or produces it in insufficient amounts, resulting in abnormally high blood sugar levels. Diabetics cause several complications, including metabolic disorders of carbohydrate, lipid, and protein in the body, as well as vasculopathies [1, 2]. The predominance of diabetes is higher in men than women [3]. It is estimated that, the global diabetes prevalence will be rising to 10.2% (578 million) by 2030 and 10.9% (700 million) by 2045 [4]. Diabetes poses a significant challenge to the health-care system in poor nations since it is a life-long disease that necessitates daily treatment and family participation. According to a recent epidemiological study in Bangladesh, the prevalence of diabetes mellitus is higher among the elderly [5]. Another study revealed that males are more affected than females in urban areas, but this scenario is reversed in rural areas [6]. The average annual cost of diabetes care in Bangladesh is US$864.7 per patient, which is significantly higher than many other countries [7]. Exercise, diet and weight management remain important and effective ways to improve glucose homeostasis. Aside from side effects, treatments with drugs are not always satisfactory to avoiding late-stage diabetic complications and maintaining euglycemia. The existing therapeutic agents are still suffering from severe limitations. Health care professionals are increasingly looking for medicinal herbs with antihyperglycemic properties as an alternative treatment option for diabetic patients [8]. Many traditional plant treatments are a hidden treasure trove of effective natural diabetic products.

*Momordica dioica* Roxb. is a member of the Cucurbitaceae family and the genus Momordica and found primarily in Africa and Asia [9]. The fruit of the plant are generally used as green vegetables with wide range of medicinal properties. The fruits have hepatoprotective, diuretic, alexiteric stomachic laxative properties, and the leaves have antihelminthic and aphrodisiac properties [10]. The root juice act as a stimulant, astringent, and antiseptic [11]. However, there is no evidence that *M. dioica* fruits have anti-diabetic properties, even though some researchers claim that *M. dioica* fruits have hypo/anti-hyperglycemic properties [11, 12, 13, 14, 15]. It was also reported to have antioxidant activity [16, 17], antimicrobial activity [18, 19], hepatoprotective activity which supports our experiment [16, 20, 21, 22], Reno protective activity [15, 23], analgesic and anti-inflammatory activity [24, 25].

*M. dioica* was selected as it is one of the usual vegetables taken by local people and its wider practices in rural areas for different treatments. Also, various research findings pointing out the potentialities of *M. Dioica* from different perspective like plant parts, animal model or standard drug [10, 13, 21, 26, 27]. The present work was therefore undertaken to explore the antidiabetic properties (glucose homeostasis, lipid profiles, serum insulin, hepatic glycogen content) of *M. dioica* ethanolic extract on type 2 diabetic model rats. This study's finding may prove constructive in the formulation of novel antidiabetic drugs and herbal industries for identification, characterization, and standardization of *M. dioica* fruits.

## 2 Materials and methods

### 2.1 Materials

Streptozocin was purchased from Sigma Aldrich Inc., St Louis, MO, USA. Glibenclamide was provided from Square Pharmaceuticals Ltd., Bangladesh., Tween 20 Acetic acid, Ethanol, Ferric Chloride, Picric acid, Hydrochloric acid, Potassium ferrocyanide were purchased from the Merck, Germany. All other reagents were analytical graded.

### 2.2 Plant specimen collection and authentication

The unripen fresh fruits of *M. dioica* were collected from commercially available sources from Khulna district, Bangladesh (Geographical coordinates 22.8020° N, 89.5330° E). The plant sample was identified with an accession number of 38590 by the Bangladesh National Herbarium (BNH) in Dhaka, Bangladesh.

### 2.3 Plant extraction preparation

Fresh unripen fruits (2.5 kg) were washed thoroughly with distilled water after collection and then finely chopped. After that, the chopped fruits were air dried evenly at room temperature and then coarsely ground into fine powder (210 gm) mechanically (Panasonic Mixer Grinder MX AC210). Finally, this dried powder portion was extracted using 70% (v/v) 1.5 L ethanolic solvent according to standard protocol [28]. The 70% ethanolic solvent was used due to its higher polarity than pure ethanol and more bioactive compounds were detected. Whatman filter paper (grade 42, Ashless) was used to filter this extract after it was filtered with a thin, clean cloth. A rotary evaporator (EYELA, USA; HEYDOLPH, Germany) set to 45 °C was used to evaporate the ethanolic solvents from the extracted solutions, yielding a semi-solid solution. Furthermore, this solution was kept in a freeze drier (HETOSICC, Heto Lab Equipment, Denmark) at -55 °C to evaporate all ethanolic solution and unwanted water portions from the crude extracted solution in order to obtain our desired plant extraction. The amount of yield extraction (7.38%) was carried by using the equation as bellow (Eq. (1)):

(1) $$Yield\;value=\frac{Wt.\;of\;the\;dry\;extract*100}{Wt.\;of\;the\;dry\;plant}$$

### 2.4 Phytochemical analysis

To determine the quantitively phytochemical compounds in ethanolic plant extract, 3 g of freshly prepared plant extract was boiled in 30 mL warm water for 5–10 min in a water bath and then filtered. To confirm the presence of phytochemicals in plant extracts, various analytical methods were used, including Mayer's and Hager's test for alkaloids, Benedict's and Fehling's tests for carbohydrate, alkaline reagent test and lead acetate test for flavonoids, gelatin test for tannins, froth test for saponins, and ferric chloride bioactive compounds test for phenols [29].

### 2.5 Preparation of animals for experiment

In our current study, we used the Streptozocin (STZ) induced type 2 diabetic rat model. The type-2 model rat was prepared using slide modification as previously described [30]. In brief, 48-hour-old rat pups were subcutaneously injected with 90 mg/kg body weight doses of STZ in a citrate buffer (0.1M, pH 4.5) solution [31]. The pups grew up in the animal house with their mother and ad libitum food, maintaining a natural day-night cycle at 22 ± 5 °C and 40–70 % humidity. Following 3 months of STZ injection, when the male rats had become 150–210 ​g, their blood glucose levels were tested by the oral glucose tolerance test (OGTT). The rats were chosen for further testing because their fasting blood glucose (BG) levels were greater than 7.00 mmol/L (Fasting BG > 7.00 mmol/L considered as a diabetic model). Before collecting fasting blood and measuring the fasting blood glucose in the rat, the rat was anesthetized with diethyl-ether. To avoid the influence of circadian rhythms, the experiments were conducted in the early morning between 7:00 and 7:30 a.m.

### 2.6 Ethical statement

All animal experiments were carried out in accordance with the National Research Council (US) Committee's 'Guide for the Care and Use of Laboratory Animals,' 8th edition [32]. The Institutional Ethical Committee for Animal use was approved by the (Ethical review committee, Bangladesh University of Health Sciences, Dhaka, Bangladesh) granted ethical approval (Memorandum: BUHS/EA/SP/16/001).

### 2.7 Design of experiment

After three months of STZ inoculation and based on fasting blood glucose test results, the diabetic rats were randomly divided into three groups, each with seven rats: Group-1: water treatments, Group-2: glibenclamide treatments, and Group-3: tested sample treatments. The water treated group was fed at the doses of 10 mL/kg BW was considered as a positive control group, glibenclamide a well-known anti-diabetic drug treated group was fed at the doses of 5 mg per 10 ml of solvent (9.9 ml H_2_O + 0.1 ml Tween 20)/kg BW which considered as the negative control group, and the tested samples (ethanolic extract of dried fruits of *M. dioica*) was feed at the doses of 1.25 g per 10 ml of water/kg BW [33, 34]. All the experimental groups were in the same condition, and their body weights were measured at regular intervals using weight scale machines. The oral administered experiments were carried out for a total of 28 days, with fasting serum glucose levels measured on the 0^th^, 14^th^, and 28^th^ days of the study. Under the fasting condition, blood samples were collected by amputation of the tail tip using diethyl ether anesthesia on the 0^th^ and 14^th^ day to measure serum glucose, total cholesterol, triglyceride, HDL, LDL and serum insulin. The tail was immersed in warm water (about 40 °C) for approximately 20–30 s prior to amputation for vasodilation. To avoid hemolysis, 0.2 ml of blood was carefully collected in the Eppendorf tube after cutting the tail tip with surgical scissors. The rat's blood was collected from the heart through cardiac puncture on the 28^th^ day after cervical dislocation, and the liver was collected to measure liver glycogen concentration.

### 2.8 Analytical methods

The Glucose Oxidase (GOD-PAP) method was used to measure the serum glucose and quantified by using a micro-plate reader (Bio-Tec, ELISA) [1]. The Enzymatic colorimetric method autoanalyzer (Randox Laboratories Ltd., UK) was used to measure total serum cholesterol (TC) (Cholesterol Oxidase/Peroxidase, CHOD-PAP), triglycerides (TG) (GPO PAP), and serum lipid (HDL-C) respectively [1]. Serum insulin was measured by using the enzyme-linked immunosorbent assay (ELISA) method according to the manufacturer's instructions (Crystal Chem Inc., Downers Grove, IL, USA). The absorbance of colorimetric solutions such as insulin was measured by using microplate reader (Bio-Tek EL-340, USA). Low density lipoprotein (LDL-C) levels were calculated using Fried Ewald's formula [35], whereas liver glycogen levels were estimated using the Anthrone-sulphuric acid method [1].

### 2.9 Statistical analysis

All experimental data was analyzed by using a Statistical Package for Social Science (SPSS) software (SPSS Inc., Chicago, Illinois, USA). The statistical analysis was performed by using one way ANOVA and Dunnett multiple test methods. The mean ± SD or as Median (Range) and the limit was calculated using Windows version 12 (SPSS Inc., Chicago, Illinois, USA). The limit of significance was set at p < 0.05.

## 3 Results and discussion

### 3.1 Phytochemical analysis

The results of the phytochemical analysis of dried fruits of *M. dioica* indicated the presence of saponin, flavonoid, phenol, alkaloid, carbohydrates and terpenoid like phytoconstituents (Table 1). Saponin type phytoconstituents are more likely responsible for antidiabetic properties in cells [36, 37, 38]. As a result, because chemical isolation was not performed in this investigation, the exact mechanism of action of the extract could not be determined. In fact, our study was supported by previous studies because the extract was only devoid of tannin [36, 37, 38].

**Table 1:** Phytochemical analysis of the *M. dioica* fruits extract.

Serial no	Phytochemicals	*M. dioica*
1	Tannin	-
2	Saponin	++
3	Alkaloid	+
4	Flavonoid	++
5	Phenol	++
6	Terpenoid	++
7	Carbohydrate	++

(++) = Presence, (+) = Trace, (-) = Absence.

### 3.2 Effect on the body weight (BW)

**Figure 1:**
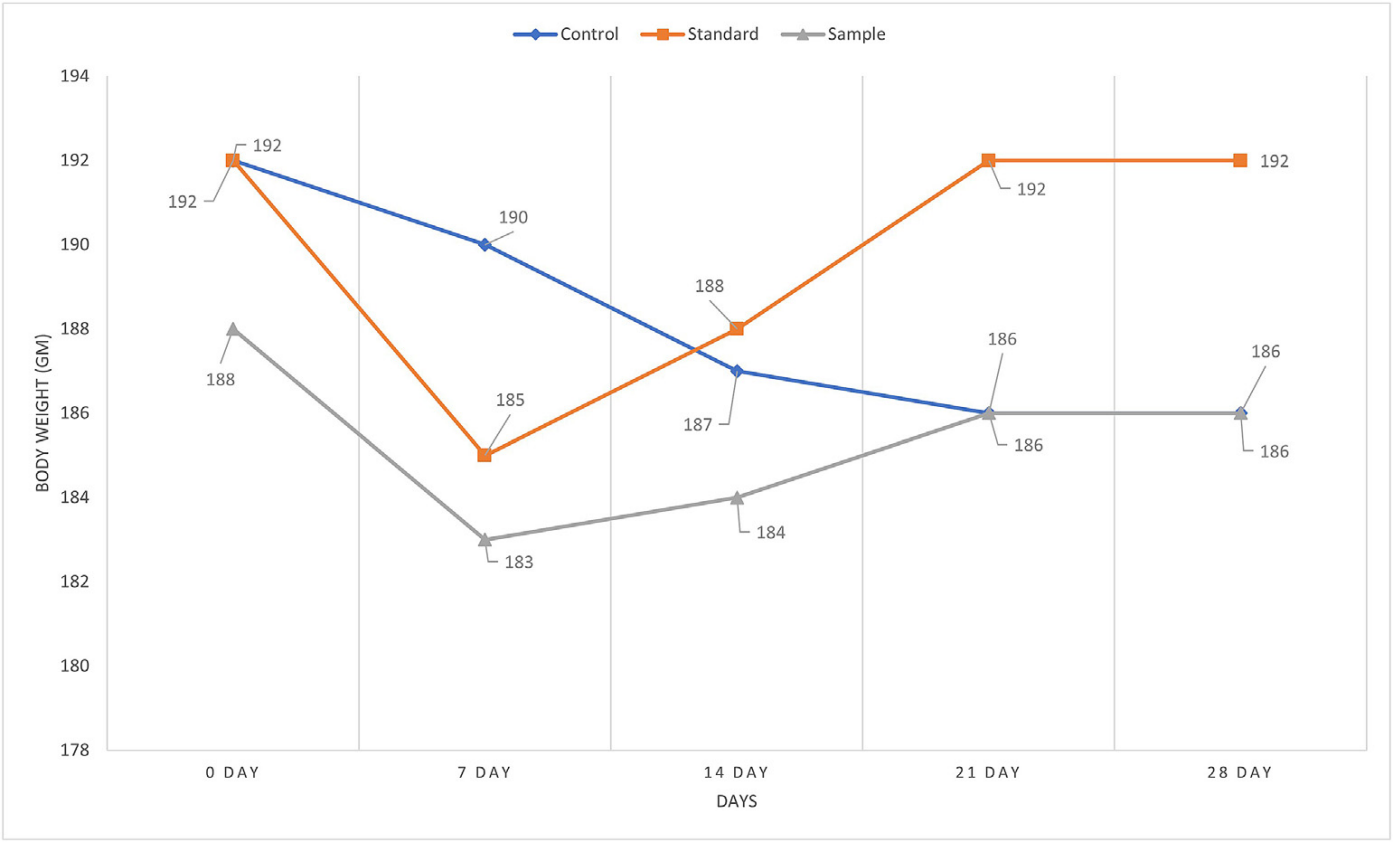
*M. dioica* extract effects on body weight in type 2 diabetic model rats.

After 28 days of meticulous observation, we found all the groups (control, standard and sample) had a proclivity to lose body weight at initial stages with higher significant value (p = 0.0002 for standard and p = 0.0001 for sample). This might be a transitory loss of appetite due to the stress caused by the gavage feeding. So, it took almost 7 days to get used to forced feeding. Increased catabolic reactions resulting in muscle wasting may result in diabetic rats having a lower body weight [39]. Because of the body weight of rats was observer regularly so that; the changes can be visualized consecutively in Figure 1. The sample group changed the body weight from the 0^th^ day with 188 ± 16 ​g up to the 28^th^ day with 189 ± 24 ​g. Later, standard and sample were maintained to balance the body weight returning to original values. The rats' body weight did not alter much after 28 days of extract feeding, according to the experiment. It was discovered that an ethanol extract of *M. dioica* has no influence on depot fat breakdown, which is a common concern in diabetes mellitus, based on body weight readings. The same observation was established by previous studies [12, 14, 15]. The outcomes indicated that the tested samples hadn't any significant impact on fat degradation.

### 3.3 Effect of *M. dioica* extract on fasting serum glucose level

**Figure 2:**
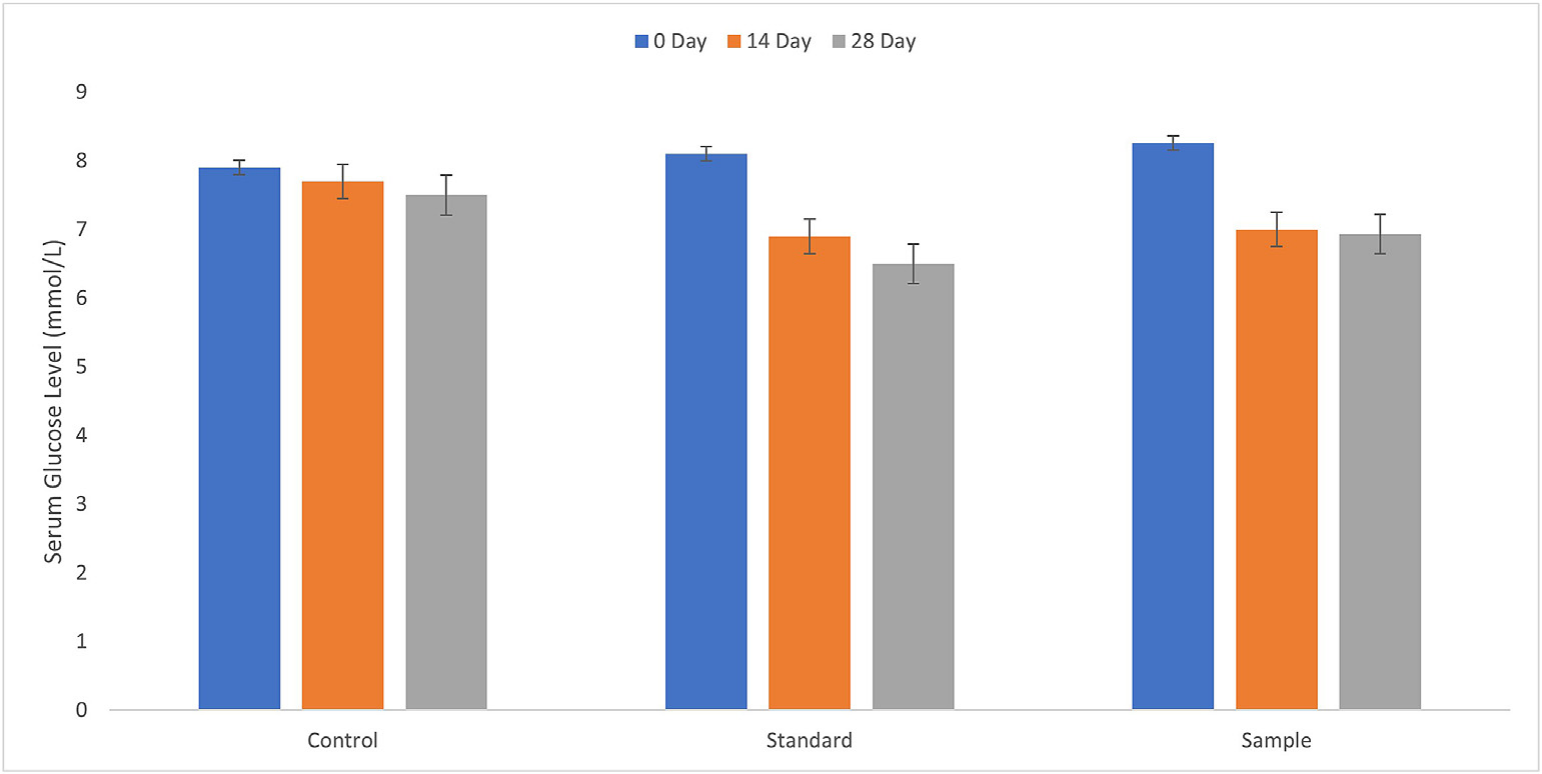
The effect of *M. dioica* extract on type 2 diabetic model rats' fasting serum glucose levels.

On the experimental time, fasting serum glucose (FSG) levels in type 2 diabetic model rats from different experimental groups were nearly identical (Figure 2). This study revealed that ethanol extract of *M. dioica* produced a hypoglycemic effect (from 8.26 ± 0.65 to 7 ± 0.24 mmol/L; *p* = 0.587) in the postprandial state on the 14^th^ day. However, on the 14^th^ day, the sample group showed a decline of the fasting serum glucose level and continued to reduce until the 28^th^ day (from 7 ± 0.24 to 6.93 ± 0.61 mmol/L; p = 0.55). The extract opposed the rise of glucose, although for *M. dioica* the value was not significant (*p* = 0.047), yet in addition, the plant extract influenced the fasting serum glucose level. After 28 days of feeding, *M. dioica* reduced fasting serum glucose levels in type 2 diabetic rats. Our outcomes are consistent among specialists [12, 14, 15].

### 3.4 Effect of *M. dioica* extract on glucose homeostasis

**Figure 3:**
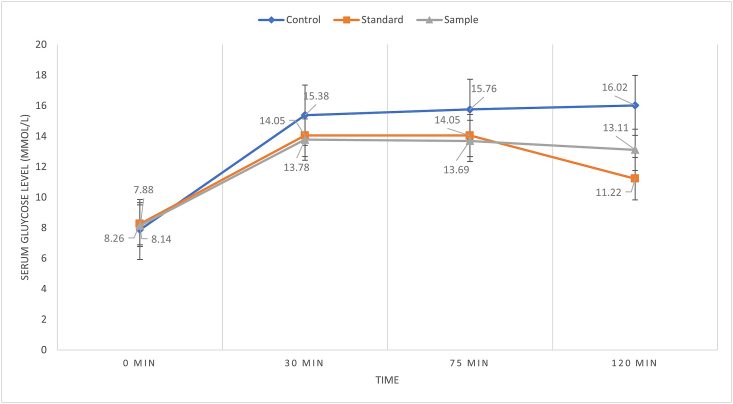
*M. dioica* extract impact on blood glucose levels of T2DM rats on the 0^th^ day.

To evaluate the effect of *M. dioica* on glucose metabolism, an oral glucose tolerance test was performed on the 0^th^, 14^th^ and 28^th^ days of the experimental period. At the initial day, when glucose was administered along with extracts, serum glucose levels were increased rapidly from 8.14 ± 0.50 to 13.78 ± 2.58 mmol/L within 30 min (Figure 3). After 75 min, identical percent of glucose level was observed (around 69%) for the standard but slightly decreased (1%) in the sample groups. Finally, after 120 min the glucose level was significantly decreased both by the standard and sample groups (*p* = 0.0014 for the standard and *p* = 0.0038 for the sample).

**Figure 4:**
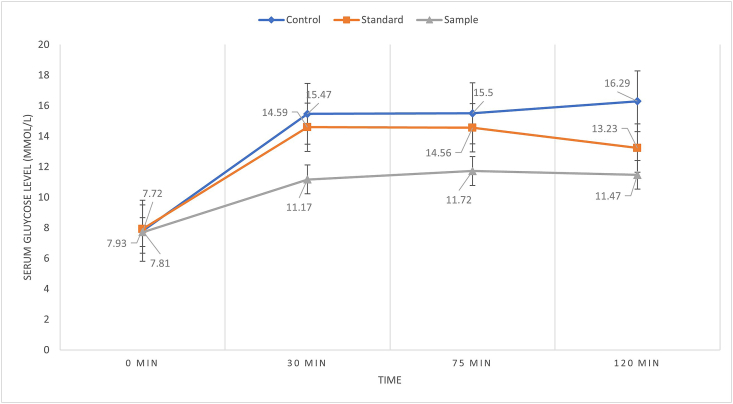
*M. dioica* extract impact on blood glucose levels of T2DM rats on the 14^th^ day.

In the Oral Glucose Tolerance Test (OGTT), after 14 days of consecutive feeding of the sample, serum glucose level was increased after 120 min in all treated groups except for the sample group (Figure 4). Here, the sample exhibited a significant (p = 0.0015) blood glucose lowering effect after 120 min compared to the control (16.29 ± 1.03 mmol/L) and standard (13.23 ± 2.21 mmol/L).

This study represented that ethanolic extract of *M. dioica* produced a significant (p = 0.0015) hypoglycemic effect in the postprandial state on the 14^th^ day. *M. dioica* extract increased the postprandial glucose level (11.47 ± 2.21 mmol/L) significantly at 120 min when compared to the control group (13.23 ± 1.03 mmol/l). The plant extract also altered postprandial serum glucose levels, according to the findings of the current investigation. After 28 days of feeding, *M. dioica* dramatically reduced fasting serum glucose levels in type 2 diabetic rats. Our findings were also supported by the findings of other researchers [12, 14, 15].

### 3.5 Effects of *M. dioica* extract on lipid profiles

**Figure 5:**
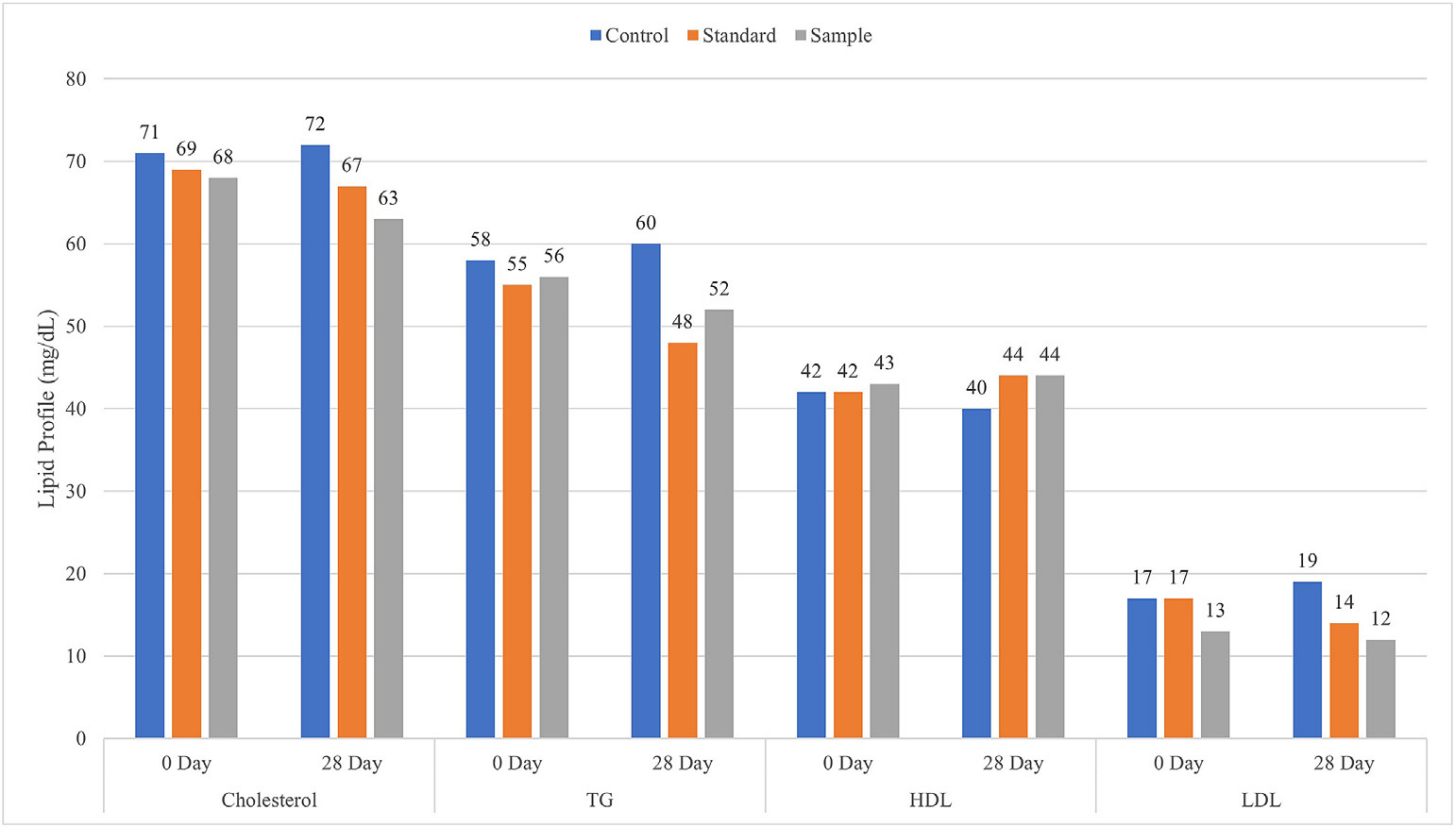
Lipid profile graphs of type-2 diabetic model rats effected by *M. dioica* extract.

Persistent hyperglycemia is now well accepted as a significant cardiovascular risk factor in type 2 diabetic individuals [40]. The link between hyperglycemia and lipid changes in diabetic patients reveals a significant risk of cardiovascular problems. Notwithstanding glycemic control, treatment of hyperlipidemia likewise results in noteworthy micro and macrovascular illnesses in people with type 2 diabetes. The noticeable effect of *M. dioica* on the lipid profile of type 2 diabetic model rats presented in Figure 5. Treatment of diabetic model rats for 28 days with *M. dioica* extract brought down serum cholesterol and triglyceride level significantly by 7% (p < 0.0001). These discoveries bolster the past reports of the viability of this plant to reduce lipid abnormalities along with the treatment of diabetes [13, 14, 26, 41]. The *M. dioica* diet can inhibit the absorption of cholesterol and promote the excretion of cholesterol and bile acid into feces [42]. Our study also depicts that the level of HDL had been increased, which is referred to as “good” cholesterol (Figure 5). In another study, diabetic rats treated with protein extract from the fruit pulp of *M. dioica* showed an increment of HDL at a significant level [26], which supports our findings. Moreover, we found that the bad cholesterol-LDL was also diminished by the dose of *M. dioica.* This data is also concordant with others [14, 26, 42].

### 3.6 Effects of *M. dioica* extracts on the serum insulin of type 2 diabetic model rats

**Figure 6:**
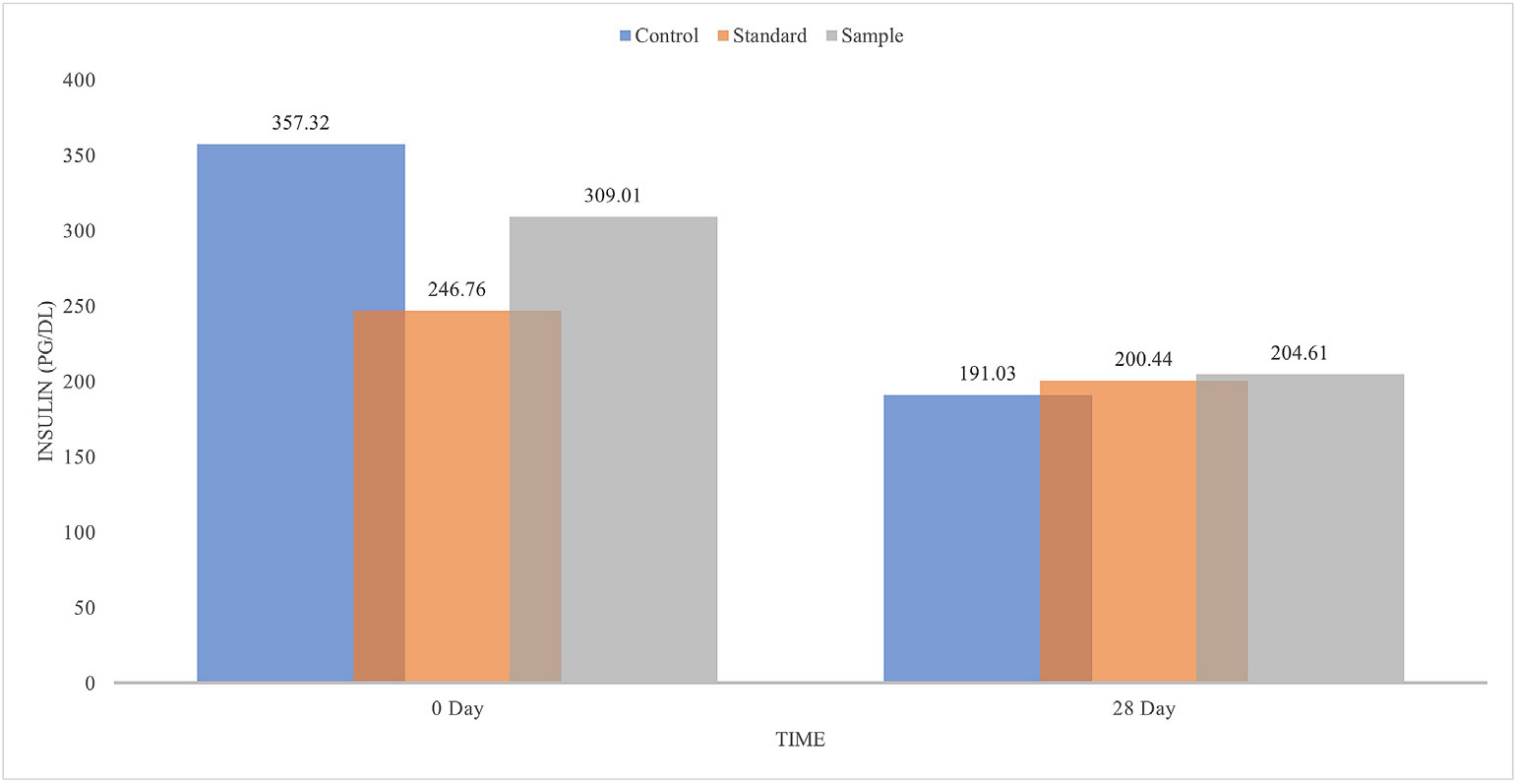
Serum insulin level of type-2 diabetic model rats effected by *M. dioica* extract.

Flawed insulin secretion is a major problem related to diabetes mellitus. Drugs like sulfonylurea stimulates insulin release from the pancreatric β-cells are broadly used for the management of type-2 diabetes [43]. The prominent effect of *M. dioica* ethanol extract on the insulin level of different groups of type 2 diabetic model rats showed in Figure 6. After 28 days of treatment, the insulin levels were found to be decreased in all groups. Around 54% of serum insulin level was decreased by the control group, whereas standard and sample were reduced 77% and 44% respectively. Therefore, it can be estimated that *M. dioica* fruit extract generates a hypoglycemic effect by inducing the secretion of insulin from the pancreas. Similar kind of study results were found but with different plant extracts [44]. It has been observed by other groups that *M. dioica* extract can induce stimulation to surviving pancreatic β-cells to release more insulin [14]. In another study the same group concluded that the route of insulin secretion by *M. dioica* extract is independent of K-ATP channels of β-cells [41]. Meanwhile, saponin isolated from the fruits of *M. dioica* revealed that it has the potential to reverse beta cell degeneration [37, 38].

### 3.7 Effect of *M. dioica* extract on hepatic glycogen content of type 2 diabetic model rats

Hyperglycemia, due to diabetes, it leads to the production of free radicals that are associated with the development of diabetic complications [45]. Free radicals also cause damage to the liver tissues. Several other papers have shown that different parts of *M. dioica* plant have scavenging activity, so it helps to protect the liver and prevents the development of fatty liver through the inhibition of intestinal lipid absorption [16, 42, 46].

The level of liver glycogen may be the most accurate indicator of a medication's hypoglycemic effect. This shows that by increasing glycogenesis, peripheral free glucose is stored as glycogen in the liver [47, 48]. Table 2 shows the effects of *M. dioica* on the hepatic glycogen level of type 2 diabetes model rats after 28 days of chronic therapy. Liver glycogen content was increased by 14% through the treatment of extract, and the accretion was statistically significant (p < 0.0005). It's possible that *M. dioica's* hypoglycemic effect is due in part to an increase in glucose uptake for glycogen formation through improved glycogenesis.

**Table 2:** Effect of *M. dioica* extract on hepatic glycogen content of type 2 diabetic model rats.

Group	Glycogen (mg/g) [M±SD]
WC (n = 7)	7.14 ± 4.48 (100%)
Glc (n = 7)	16.03 ± 4.82 (128%)
MDEtE (n = 7)	15.85 ± 3.98 **(114%)**

## 4 Conclusions

In type 2 diabetes model rats, ethanol extract of *M. dioica* improved glycemic and lipidemic conditions and is ready to improve the diabetic state. According to our findings the fasting serum glucose, cholesterol and triglyceride levels were noticeably reduced by the treatment with *M. dioica* fruit's ethanolic extract. There are limited clinical evidence for the traditional and local uses of this fruit in various treatments, and there are no panoptic reports on the primary active Phyto-constituents of *M. dioica* ethanolic extract. As a result, determining which phytochemicals are responsible for anti-diabetic activity is difficult. Further research into the molecular mechanism and the isolation of the compound responsible for this propensity may lead to the development of a new antidiabetic agent.

## Declarations

### Author contribution statement

Md. Mynul Hassan, Shihab Uddin: Performed the experiments; Wrote the paper.

Amrita Bhowmik: Analyzed and interpreted the data; Conceived and designed the experiments.

Begum Rokeya, Ayesha Ashraf: Conceived and designed the experiments; Contributed reagents, materials, analysis tools or data.

Md. Mahmodul Islam: Analyzed and interpreted the data; Wrote the paper.

### Funding statement

This work was supported by the Asian Network of Research on Anti-Diabetic Plant (ANRAP) & International Program in the Chemical Sciences (IPICS).

### Data availability statement

Data will be made available on request.

### Declaration of interests statement

The authors declare no conflict of interest.

### Additional information

No additional information is available for this paper.
